# Supplementary material for: The Cactus (Opuntia ficus-indica) Cladodes and Callus Extracts: A Study Combined with LC-MS Metabolic Profiling, In-Silico, and In-Vitro Analyses
Source: Antioxidants (Basel). 2023 Jun 23;12(7):1329. doi: 10.3390/antiox12071329 (PMC10376840; doi:10.3390/antiox12071329)
Supplement: Supplementary file 1 [file antioxidants-12-01329-s001.zip › antioxidants-2415934-supplementary-7.4.pdf]

Supplementary data

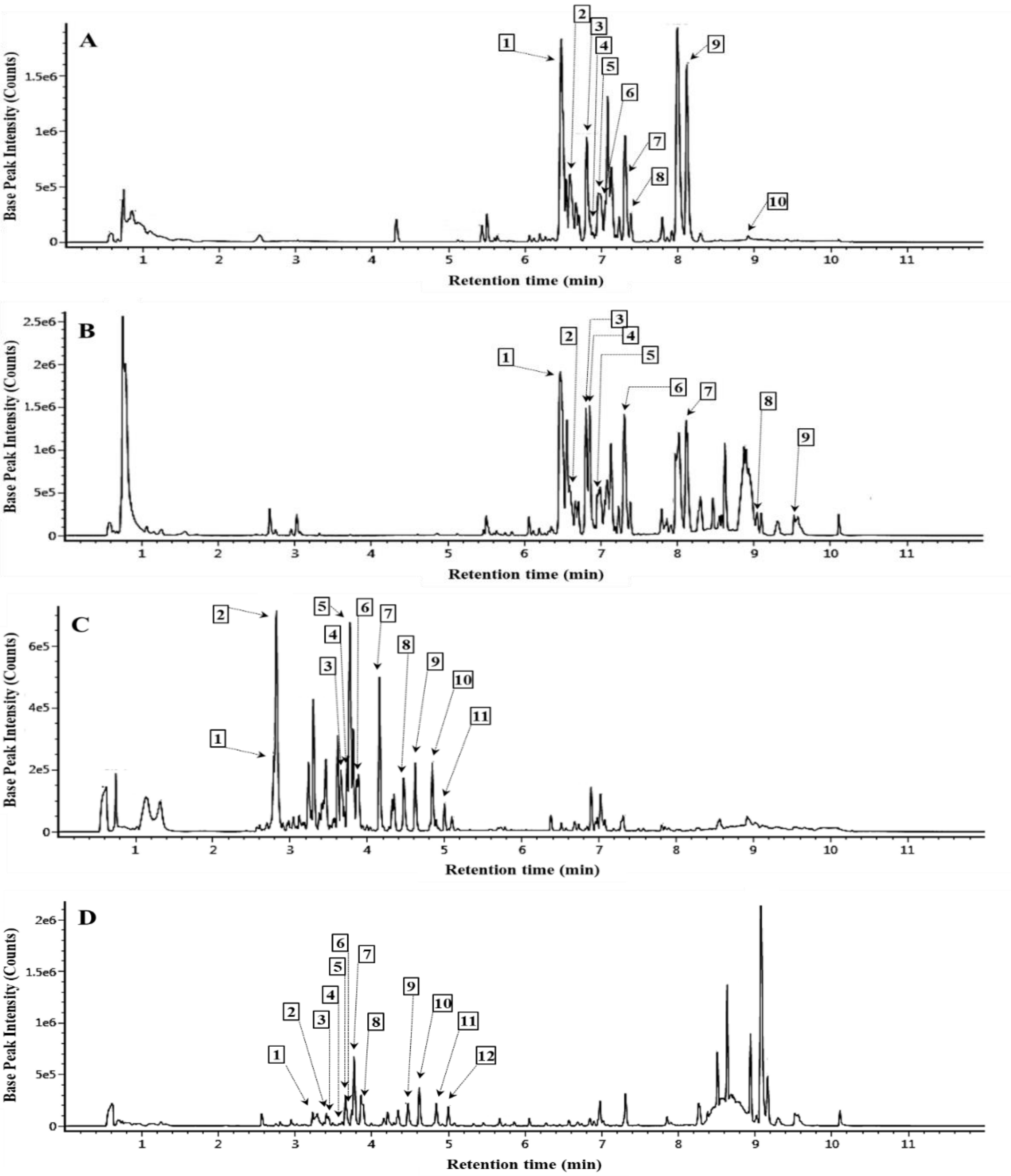

Figure S1. UPLC-QTOF-MS metabolite component profile (A) negative ion detected in OFC extract, (B) positive ion detected in OFC extract, (C) negative ion detected in OF extract, and (D) positive ion detected in OF extract. Compound information for each peak number in these chromatogram is shown in Table S1 and S2.

Table S1. Identification of metabolite components in OFC extract.

No.	Metabolite Class	t_R (min)	Tentative identification	Molecular Formula	Molecular Weight	Error (ppm)	MS/MS Fragments
Adducts ion [M-H]⁻							
1	Triterpenoid	6.48	Poricoic acid H	$C_{31}H_{48}O_5$	499.3430	−0.24	499/437/286/153
2	Triterpenoid	6.59	Colossolactone VII	$C_{33}H_{50}O_7$	557.3480	0.62	557/539/483
3	Triterpenoid	6.81	Poricoic acid A	$C_{31}H_{46}O_5$	497.3273	−0.08	497/485/318/218/99
4	Fatty acids	6.83	LPE (18:2)	$C_{23}H_{44}NO_7P$	476.2784	−0.37	476/424/279/242
5	Steroid	6.88	Sootepin D	$C_{31}H_{48}O_4$	483.3481	−0.22	483/472/99
6	Ester	7.06	Cholesteryl hemisuccinate	$C_{31}H_{50}O_4$	485.3636	0.15	485/396/297/265
7	Triterpenoid	7.31	Poricoic acid C	$C_{31}H_{46}O_4$	481.3324	−0.06	481/470/277/220/99
8	Fatty acids	7.31	LPA (18:2)	$C_{21}H_{39}O_7P$	433.2365	−0.89	433/311/152
9	Steroid	8.12	Pachymic acid	$C_{33}H_{52}O_5$	527.3733	2.25	527/515/99
10	Steroid	8.94	Eburicoic acid	$C_{31}H_{50}O_3$	469.3676	2.99	469/458/382/313
Adducts ion [M+H]⁺							
1	Triterpenoid	6.47	Polyporenic acid C	$C_{31}H_{46}O_4$	483.3470	1.95	483/465/381/327/291
2	Antifungal	6.59	Leptomycin B	$C_{33}H_{48}O_6$	541.3524	1.93	541/523/449/385/165
3	Triterpenoid	6.81	Corticatic acid D	$C_{31}H_{44}O_4$	481.3311	2.48	481/455/325/165
4	Fatty acids	6.83	LPE (18:2)	$C_{23}H_{44}NO_7P$	478.2927	2.54	478/346/337/328/310
5	Fatty acids	6.85	LPC (18:2)	$C_{26}H_{50}NO_7P$	520.3399	1.87	520/467/327/184
6	Triterpenoid	7.08	Corticatic acid B	$C_{31}H_{44}O_3$	465.3370	1.01	465/309/165
7	Triterpenoid	8.12	11,13,20,22-Hentriacontatetraynoic acid	$C_{31}H_{46}O_2$	451.3569	2.71	451/433/295/165/128
8	Fatty amide	9.04	*cis*-11-Eicosenamide	$C_{20}H_{39}NO$	310.3101	4.73	310/293/240/144
9	Fatty amide	9.20	Erucamide	$C_{22}H_{43}NO$	338.3416	3.75	338/321/303/268/196

Table S2. Identification of metabolite components in OF extract.

No.	Metabolite Class	t_R (min)	Tentative identification	Molecular Formula	Molecular Weight	Error (ppm)	MS/MS Fragments
Adducts ion [M-H]⁻							
1	Phenolic	2.79	3-Carboxy-4-hydroxy-phenoxy glucoside	$C_{13}H_{16}O_9$	315.0712	3.00	315/286/200/167/138
2	Phenolic	2.82	Piscidic acid	$C_{11}H_{12}O_7$	255.0502	3.20	255/193/165/147/107
3	Flavonol	3.66	Isoquercetin (quercetin 3-*O*-glucoside)	$C_{21}H_{20}O_{12}$	463.0871	2.42	463/433/387/300/205
4	Flavonol	3.74	Kaempferol 3-*O*-rutinoside	$C_{27}H_{30}O_{15}$	593.1497	2.47	593/551/477/345/206/164
5	Flavonol	3.77	Isorhamnetin-3-*O*-rutinoside	$C_{28}H_{32}O_{16}$	623.1598	3.17	623/537/385/300
6	Flavonol	3.88	Isorhamnetin 3-glucoside	$C_{22}H_{22}O_{12}$	477.1027	2.45	477/403/301/243/116
7	Flavanone	4.16	Eriodictyol	$C_{15}H_{12}O_6$	287.0553	2.97	287/259/243/163/116
8	Flavonol	4.47	Quercetin	$C_{15}H_{10}O_7$	301.0343	3.44	301/233/178/116
9	Flavonol	4.62	Isorhamnetin	$C_{16}H_{12}O_7$	315.0500	3.13	315/300/271/243/116
10	Flavonol	4.85	Kaempferol	$C_{15}H_{10}O_6$	285.0390	5.13	285/271/204/170
11	Flavone	5.00	Hispidulin	$C_{16}H_{12}O_6$	299.0547	4.69	299/284
Adducts ion [M+H]⁺							
1	Flavone	3.23	Typhaneoside	$C_{34}H_{42}O_{20}$	771.2345	1.10	771/625/433
2	Flavonol	3.40	Isorhamnetin 3-sophoroside-7-rhamnoside	$C_{34}H_{42}O_{21}$	787.2297	0.70	787/625/459/317/302/154
3	Triterpenoid	3.43	Astragaloside	$C_{28}H_{32}O_{17}$	641.1723	0.08	641/479/317/302/220
4	Flavonol	3.58	Rutin (quercetin 3-*O*-rutinoside)	$C_{27}H_{30}O_{16}$	611.1620	−0.35	611/541/463/303/249/224
5	Flavonol	3.66	Isoquercetin (quercetin 3-*O*-glucoside)	$C_{21}H_{20}O_{12}$	465.1036	0.49	465/303/173
6	Flavonol	3.74	Kaempferol-3-*O*-rutinoside	$C_{27}H_{30}O_{15}$	595.1670	−0.28	595/449/287
7	Flavonol	3.78	Isorhamnetin-3-*O*-rutinoside	$C_{28}H_{32}O_{16}$	625.1773	0.21	625/479/317
8	Flavonol	3.87	Isorhamnetin 3-glucoside	$C_{22}H_{22}O_{12}$	479.1189	1.34	479/317/302/153
9	Flavonol	4.48	Quercetin	$C_{15}H_{10}O_7$	303.0496	4.71	303/289/198
10	Flavonol	4.62	Isorhamnetin	$C_{16}H_{12}O_7$	317.0655	3.77	317/302/198
11	Flavonol	4.84	Kaempferol	$C_{15}H_{10}O_6$	287.0547	4.99	287/166
12	Flavone	5.00	Hispidulin	$C_{16}H_{12}O_6$	301.0705	4.32	301/286

Table S3. Identification of metabolite components fragments in OFC extract.

No.	Molecular ion peak and fragmentation	No.	Molecular ion peak and fragmentation
Adducts ion [M-H]⁻		***Adducts ion [M+H]⁺***	
1.	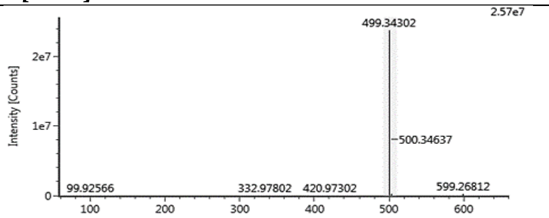	1	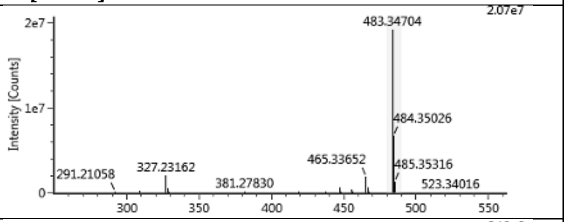
2	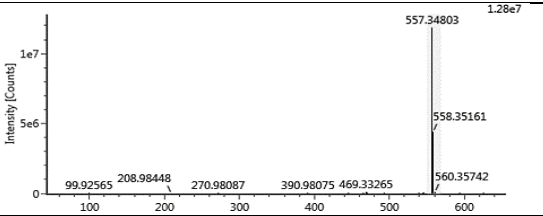	2	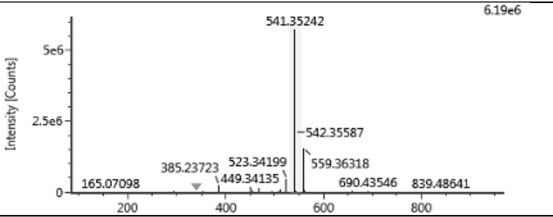
3	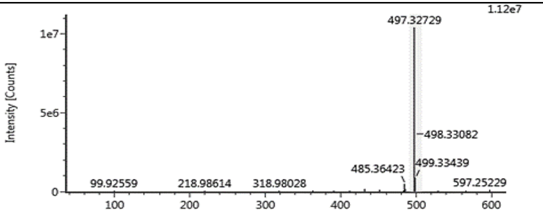	3	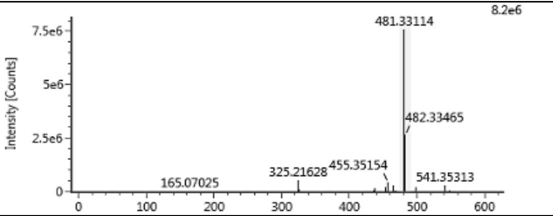
4	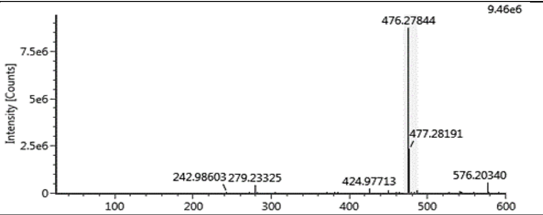	4	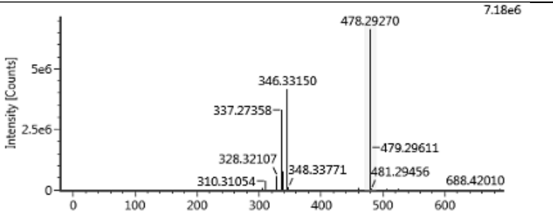
5	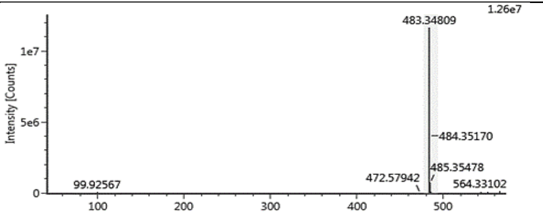	5	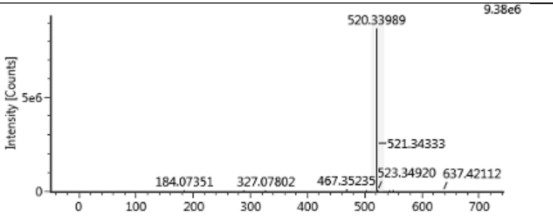
6	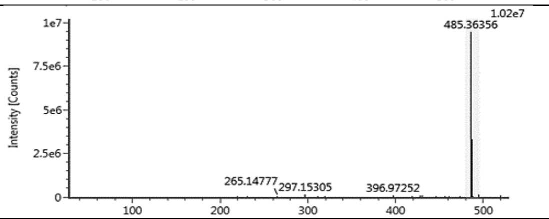	6	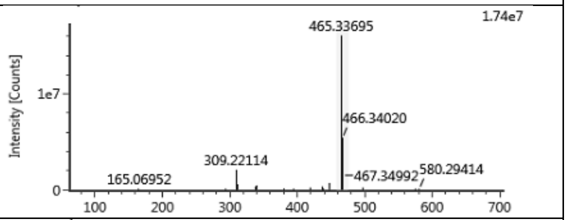
7	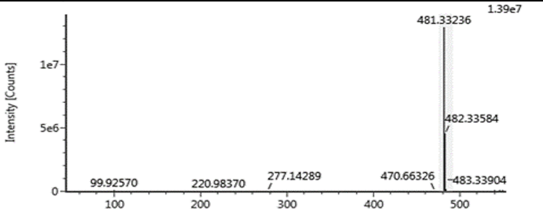	7	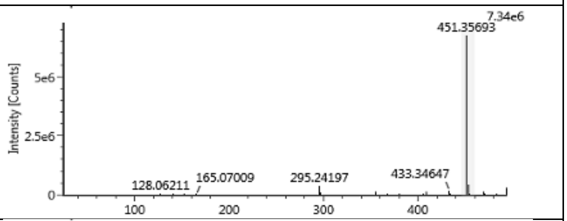
8	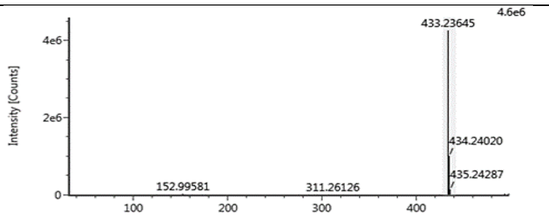	8	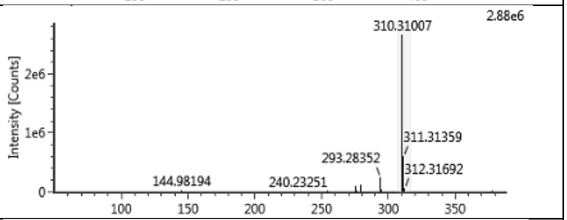
9	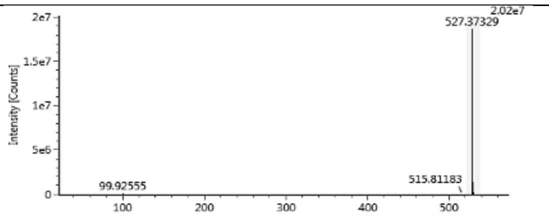	9	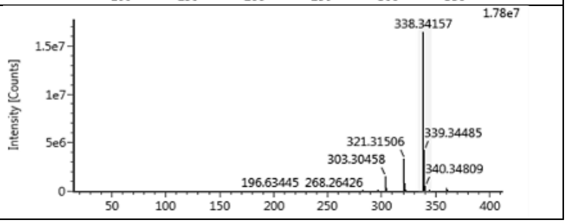

10	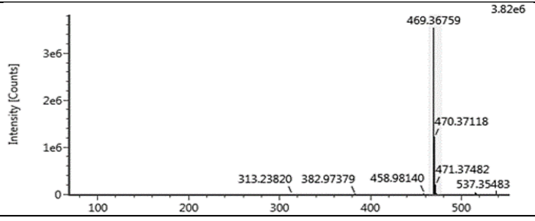		

Table S4. Identification of metabolite components fragments in OF extract.

No.	Molecular ion peak and fragmentation	No.	Molecular ion peak and fragmentation
Adducts ion [M-H]⁻		***Adducts ion [M+H]⁺***	
1.	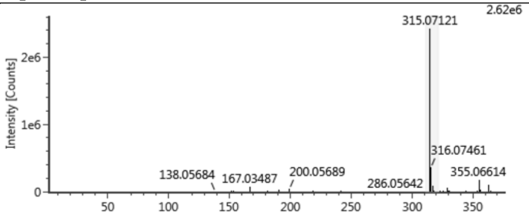	1	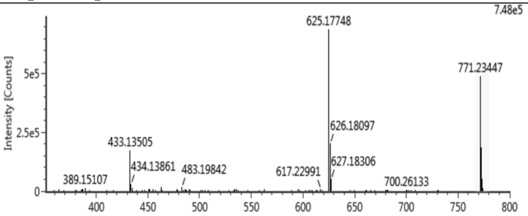
2	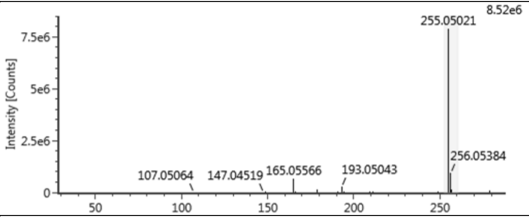	2	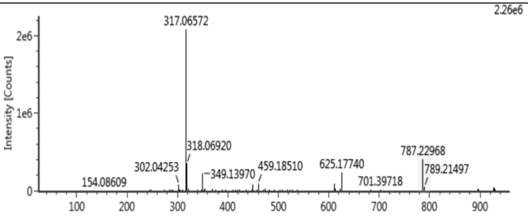
3	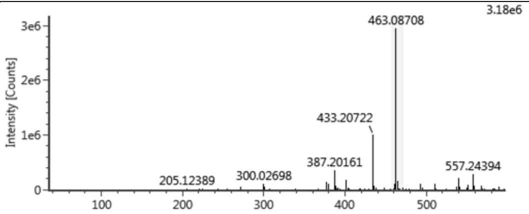	3	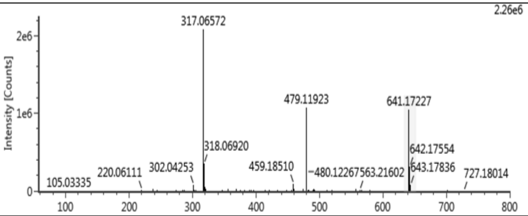
4	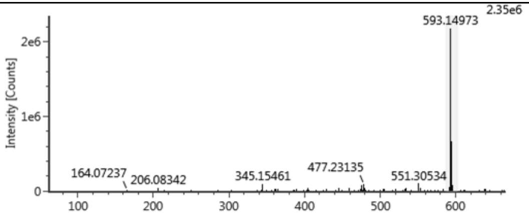	4	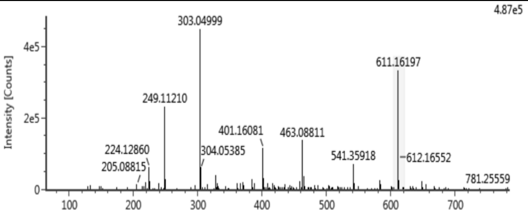
5	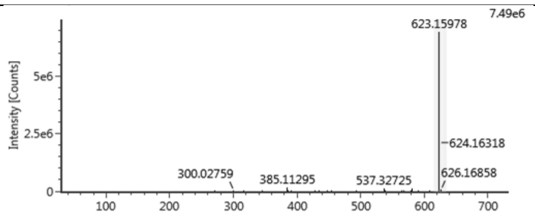	5	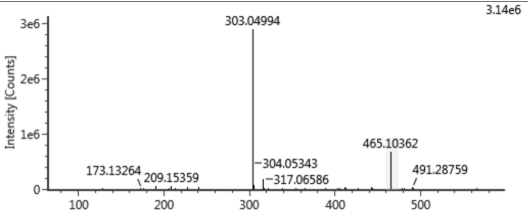
6	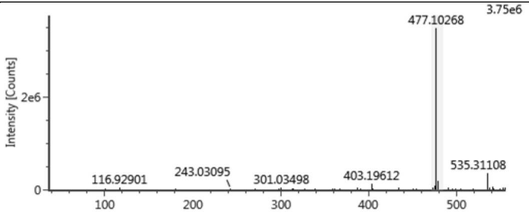	6	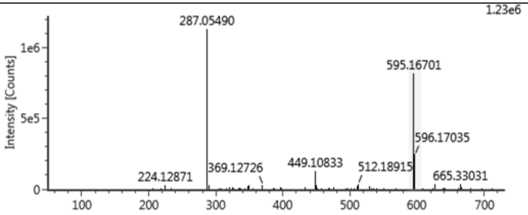
7	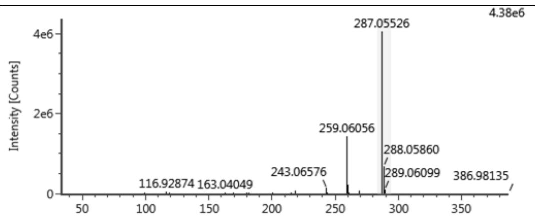	7	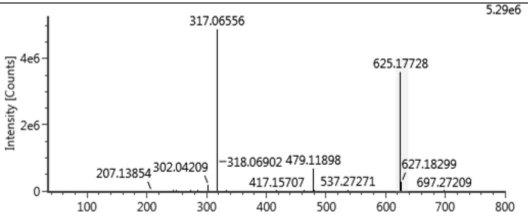

8	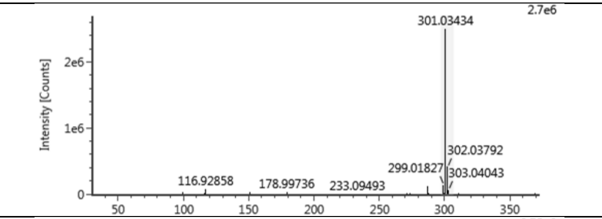	8	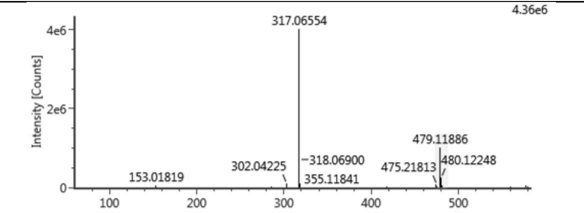
9	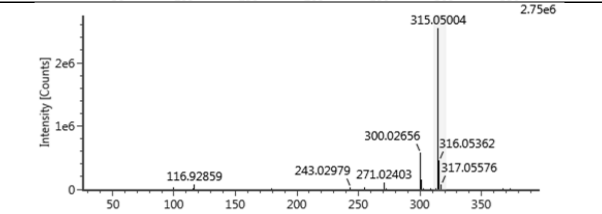	9	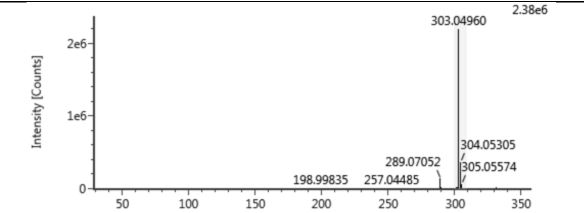
10	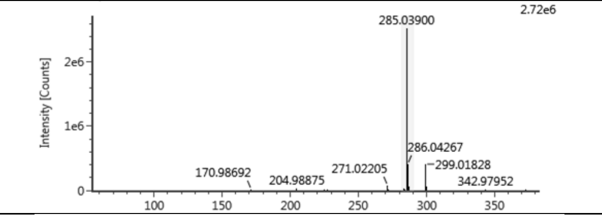	10	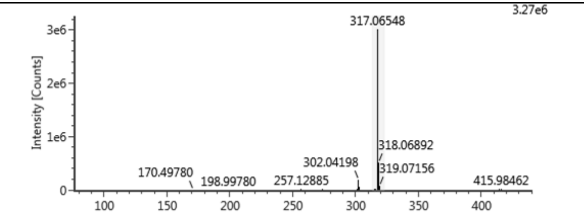
11	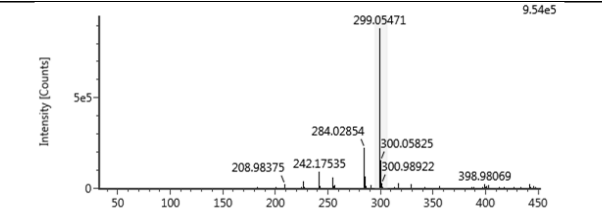	11	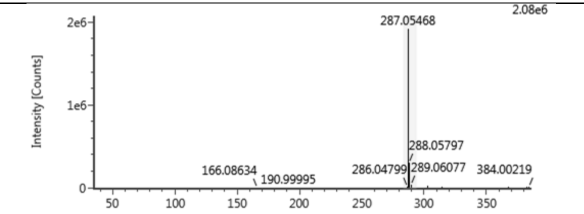
		12	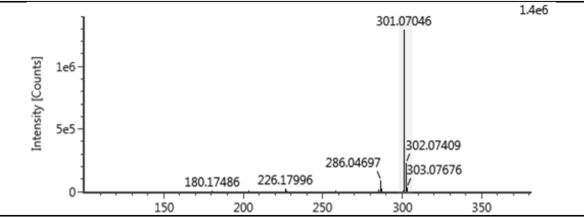

Table S5. Energy of complexes formed between selected compounds and target protein

Compound	Receptor	Toxicity	Binding energy (Kcal/mol)	pKi value (μM)	Interacting Amino Acid Residues
Selected saponin compounds from OFC extract					
Poricoic acid H	TLR4	None	−6.2	28.53	LYS-47, GLY-70
Colossolactone VII			−6.6	14.53	ALA-118, LEU-138
Poricoic acid A			−6.3	24.10	CYS-40, PRO-49, GLY-70
Poricoic acid C			−6.3	24.10	LYS-47, ILE-48, HIS-68
Poricoic acid H	MAPK	None	−8.3	0.82	GLY-110, ALA-111, ASP-112, ASP-168
Colossolactone VII			−7.4	3.77	ASN-196, SER-251, SER-252
Poricoic acid A			−8.2	0.98	GLY-110, ALA-111, ASP-168
Poricoic acid C			−7.8	1.92	LYS-53
Selected flavonoid compounds from OF extract					
Quercetin	TLR4	None	−6.5	17.20	GLN-163, PHE-165, LYS-166, GLN-188, SER-189, ASP-194
Rutin			−7.6	2.69	ILE-218, LEU-238, SER-240, ASP-243, ARG-268
Kaempferol			−6.5	17.20	GLN-163, PHE-165, LYS-166, GLN-188, SER-189, ILE-190
Isoquercetin			−7.2	5.28	GLN-142, SER-164, PHE-165, LYS-166, GLN-188, SER-189
Quercetin	MAPK	None	−7.3	4.46	TRP-197, ASP-292
Rutin			−9.0	0.25	LYS-53, LEU-104, MET-109, GLY-110, ASP-112, ASN-155, ASP-168
Kaempferol			−7.6	2.69	TRP-197, ASN-201
Isoquercetin			−8.1	1.16	LYS-53, LEU-108, MET-109, GLY-120, ASP-168
